# Absolute and Relative Morbidity Burdens Attributable to Various Illnesses and Injuries Among Active Component Members of the U.S. Armed Forces, 2024

**Published:** 2025-09-20

*MSMR* 's annual burden of disease reports are designed to provide accurate estimations of the general health status of U.S. military personnel, for prioritization of effective interventions with measurable impacts on force readiness. ^1^ In these reports, diagnoses are grouped to inform readers of the major factors and variables each year affecting health care provision within the Military Health System (MHS). Although the burden of disease within a health system can be classified into several categories, the majority of the global disease burden results from non-communicable diseases, followed by communicable diseases, maternal and neonatal diseases, nutritional diseases, and injuries. ^2^

To broadly describe the morbidity burden among active component service members (ACSMs), since 2001 *MSMR* has used a classification system derived from the Global Burden of Disease (GBD) Study. ^3 , 4^ This systematic classification, developed through a 30-year scientific effort, quantifies major diseases, risk factors, and intermediate clinical outcomes in a standardized manner, enabling comparisons between populations and health problems over time. ^5 , 6^ *MSMR* utilizes the GBD classification system in combination with an International Classification of Diseases, 10th Revision, Clinical Modification (ICD-10-CM) chapter-based system for categorizing hospitalizations and ambulatory care visits among the MHS population.

To improve the utility of this information, these classification schemes are refined by *MSMR* 's editorial staff. The major classification system for diagnoses, ICD-10-CM, features more than 68,000 separate codes. ^5^ While the ICD-10-CM is organized in logical chapters, the groupings are not optimal for articulating burdens of disease within a military population. Consequently, some re-groupings of diagnoses are necessary to achieve a meaningful depiction of the burden in the military population.

The burden of disease experienced by ACSMs—a demographic characterized by youth, good health, and a predominantly male population—is assumed to substantially differ from the burden observed for the general U.S. and global populations. This divergence is attributable to a constellation of factors, including 1) pre-accession medical screening designed to ensure physical fitness for military service, 2) mandatory periodic health assessments and screenings, which potentially lead to earlier detection of certain conditions, 3) frequent use of outpatient services for readiness-related requirements, 4) unique environmental and lifestyle factors associated with military life and training, and 5) universal access to medical care without direct financial cost. These factors, collectively, contribute to distinct morbidity burden profiles within the ACSM population.

Individuals enlist or are commissioned into the active component typically between the ages of 17 and 25 years, with almost all members ending service by age 50 years. In 2024, the largest age group within the U.S. active component was 20-24 years, followed by 25-29 years, according to Defense Medical Surveillance System (DMSS) data. Women accounted for 19.4% of the active component in 2024.

Within the military population and its unique environment, categories of illness and injury requiring hospitalization have historically differed from illness and injury categories that result in the most outpatient visits. Added requirements for military readiness are likely a major factor in outpatient health care provision, but rarely for hospitalization. The categories of medical conditions that account for the most medical encounters generally within the Military Health System may differ from those that affect the most individuals, or those that result in the most debilitating or long-lasting effects among service members. ^4^

**What are the new findings?**

Within the Military Health System in 2024, injuries, mental disorders, and musculoskeletal diseases were the major categories of medical conditions associated with the most medical encounters, greatest numbers of affected service members, and highest numbers of hospital bed days. Those three categories showed modest growth, increasing by about 0.8% compared to 2023. While reported health care encounters increased by 1.3% in 2024, the numbers of affected individuals and hospital bed days decreased by 4.4% and 2.9%, respectively.

**What is the impact on readiness and force health protection?**

The major categories of medical conditions in this report present health challenges among U.S. active component service members that can affect force readiness. Continuous health surveillance, morbidity trend analysis, and timely reporting of comprehensive summaries of the major health issues affecting the active duty force provides crucial evidence to line commanders, Military Health System leaders, and health care providers as they establish policies and priorities for effective health care management and treatment of U.S. service members.

This annual summary uses several health care burden measures to quantify the impacts in 2024 of various illnesses and injuries among members of the active component of the U.S. Armed Forces. Health care burden metrics include the total number of medical encounters, individuals affected, and hospital bed days. A consistent and comparative description of the burden of diseases and injuries, and sub-populations affected, should be an important element of health decision-making and planning processes, providing valuable information for where changes in policy or preventive emphasis may improve the medical readiness of the force. ^7^

## Methods

The population for this analysis included all individuals who served in the active components of the Army, Navy, Air Force, Marine Corps, or Space Force at any time during the surveillance period of January 1, 2024 through December 31, 2024. Each service member contributed medical records and person-time only for actual months served during the surveillance period.

All data in this analysis were derived from records maintained in the DMSS, which documents both ambulatory care encounters and hospitalizations of active component members of the U.S. Armed Forces. DMSS contains all encounters in military medical and civilian treatment facilities when reimbursed through the MHS. Encounters not routinely and completely documented within fixed military and non-military hospitals and medical clinics (e.g., during deployments, field training exercises, or at sea) were excluded from this analysis.

DMSS data for all inpatient and outpatient medical encounters of ACSMs during the surveillance period were summarized according to the primary (i.e., first-listed) diagnosis if reported with an ICD-10 code between A00 and T88, in addition to an ICD-10 code beginning with Z37 (“outcome of delivery”) or Department of Defense (DOD) unique personal history codes DOD0101–DOD0105 (“personal history of traumatic brain injury”). This year, 4 new diagnostic groups were added for analysis: pain in foot, chronic rhinitis, neoplasm of uncertain behavior of skin, and disorder of pituitary gland.

All illness- and injury-specific diagnoses, defined by ICD-10 codes, are grouped into 25 burden of disease-related categories, comprised of 157 medical conditions, based on a modified version of the classification system developed for the GBD Study. ^4^ This classification system was developed by the *MSMR* editorial staff in 2001 and is updated annually.

The GBD system groups diagnoses with common pathophysiological or etiological bases or significant DOD health policy importance. In this report, some diagnoses grouped into single categories in the GBD system (e.g., mental health disorders) were dis-aggregated to increase military relevance. In addition, injuries are classified by affected anatomical site rather than cause, as external causes of injuries are not required to be documented by providers.

The morbidity burdens attributable to various conditions were estimated based on the total number of medical encounters associated with each condition, i.e., total hospitalizations and ambulatory visits for the condition, with a limit of 1 encounter for an individual per condition each day; and numbers of service members affected by each condition, i.e., individuals with at least 1 medical encounter for the condition during the year; as well as total bed days during hospitalizations for each condition.

## Results

### Morbidity burden, by category

Provisional data indicate that affected ACSMs (n=557,980) experienced medical encounters due to injury more than any other morbidity-related category in 2024 **(Figure 1a)** . Ranking third in terms of hospital bed days, injuries accounted for about one-fourth (23.5%) of all medical encounters **(Figure 1b)** . The injury category combines ICD-10 ‘S’ (“injury”) and ‘T’ codes (“burns and poisonings”), but injuries account for about 98.1% of ambulatory encounters within the category (data not shown).

**FIGURE 1a.**
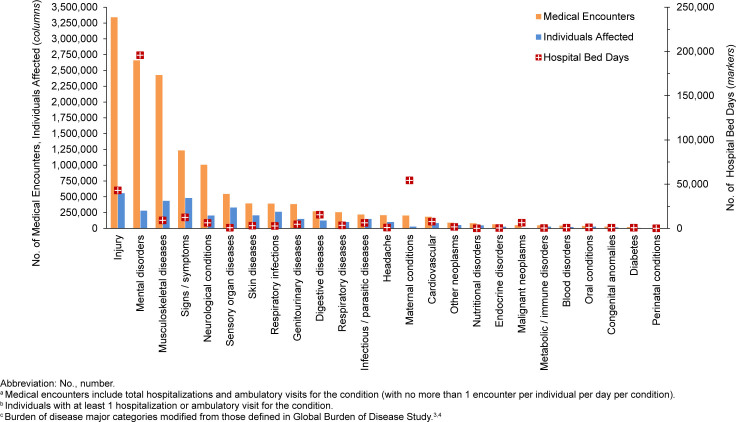
Numbers of Medical Encounters ^a^ , Individuals Affected ^b^ and Hospital Bed Days by Burden of Disease Major Category ^c^ , Active Component, U.S. Armed Forces, 2024

**FIGURE 1b.**
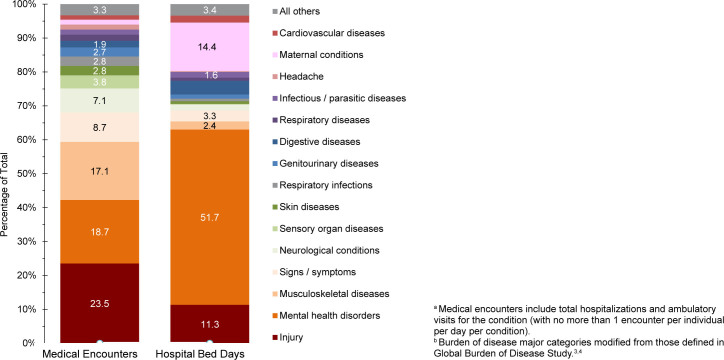
Percentage of Medical Encounters ^a^ and Hospital Bed Days Attributable to Burden of Disease Major Categories ^b^ , Active Component, U.S. Armed Forces, 2024

Mental health disorders accounted for more hospital bed days (n=195,726) than any other morbidity-related category, contributing over half (51.7%) of all hospital bed days, ranking fifth for individuals affected **(Figures 1a** , **1b)** . Together, the injury and mental health disorder categories accounted for over two-thirds (63.0%) of all hospital bed days and 42.3% of all medical encounters in 2024.

Maternal conditions (pregnancy complications and delivery) accounted for a relatively large proportion of all hospital bed days (n=54,348, 14.4%) but a much smaller proportion of medical encounters overall (n=203,467, 1.4%) **(Figures 1a** , **1b)** . As women comprised only 19.4% of the active duty force in 2024, these summary statistics understate the impact of these conditions among that group. Maternal conditions were the most frequent category for hospitalization among women in the active component.

### Medical encounters, by condition

**FIGURE 2.**
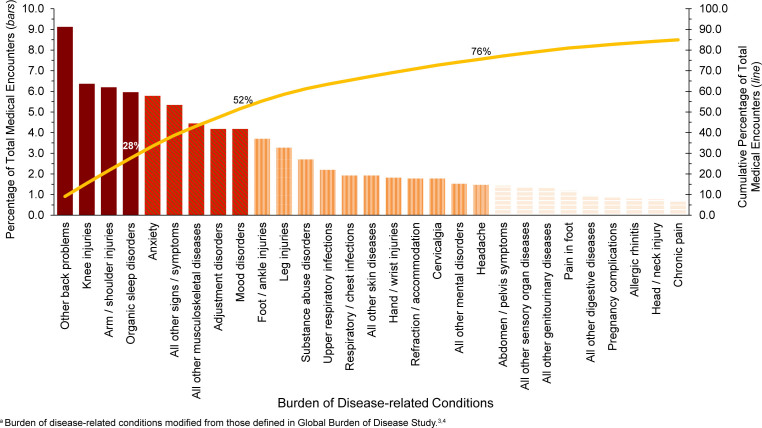
Percentage and Cumulative Percentage Distribution, Burden of Disease-related Conditions ^a^ that Accounted for the Most Medical Encounters, Active Component, U.S. Armed Forces, 2024

In 2024, almost one-third (33.4%) of all illness- and injury-related medical encounters were due to 5 medical conditions: other back problems (lower back pain, other dorsalgia), knee, arm / shoulder, organic sleep disorders (insomnia, obstructive sleep apnea), and anxiety **(Figure 2)** . Moreover, the 10 conditions associated with the most medical encounters constituted more than half (55.3%) of all illness- and injury-related medical encounters.

The categories of conditions that accounted for the most medical encounters among ACSMs in 2024 were predominantly injuries, mental health disorders, and musculoskeletal diseases. Among reported injuries, knee (6.4%), arm / shoulder (6.2%), foot / ankle (3.7%), and leg (3.3%) resulted in the most medical encounters **(Figure 2** and **Table)** . Mental health disorder diagnoses resulted most frequently from anxiety (5.8%), adjustment (4.2%), mood (4.2%), and substance abuse disorders (2.7%). Other back problems (9.1%), all other musculoskeletal diseases (4.4%), and cervicalgia (1.8%) generated the most medical encounters from musculoskeletal diseases. COVID-19 accounted for 0.2% of total medical encounters in 2024, ranked fifty-eighth, declining from 0.3% in 2023.

**TABLE 1.** Health Care Burdens Attributable to Various Diseases and Injuries, Active Component, U.S. Armed Forces, 2024

Major Category Condition ^a^	Medical Encounters ^b^		Individuals Affected ^c^		Hospital Bed Days	
	No.	Rank ^d^	No.	Rank ^d^	No.	Rank ^d^
Total	14,197,058				378,693	
**Injury, poisoning**	**3,340,649**				**42,962**	
Knee injury	903,534	2	168,709	7	892	41
Arm, shoulder injury	879,731	3	156,505	9	2,809	24
Foot, ankle injury	525,792	10	144,691	11	1,913	29
Leg injury	463,982	11	107,647	16	7,209	10
Hand, wrist injury	257,951	16	88,008	18	1,197	36
Head, neck injury	109,753	28	53,409	26	11,645	7
Back, abdomen injury	58,913	36	31,844	35	5,071	14
Unspecified injury	37,272	46	25,100	43	854	42
Other complications not otherwise specified	34,326	50	18,205	57	6,958	11
Other harm from external causes	26,528	57	16,999	58	742	48
Environmental injury / poisoning	22,909	64	16,908	59	784	45
Poisoning, non-drug	6,411	105	4,924	91	294	74
Poisoning, drugs	4,463	114	1,563	112	2,243	26
Other superficial injury	3,868	117	1,078	118	3	146
All other injuries	3,237	119	2,708	105	160	85
Other burns	1,953	126	1,194	117	188	83
Under-dosing	26	157	26	155	0	150
**Mental disorders**	**2,660,116**				**195,726**	
Anxiety	820,844	5	129,721	12	26,862	5
Adjustment disorder	593,359	8	112,119	15	32,554	4
Mood disorder	593,268	9	77,524	20	65,371	1
Substance abuse disorder	383,340	12	30,178	37	58,584	2
All other mental disorders	216,219	19	62,798	23	3,760	19
Personality disorder	20,666	68	3,734	98	2,194	27
Psychotic disorder	17,419	71	1,655	111	5,957	13
Somatoform disorder	9,984	91	3,784	97	427	65
Tobacco dependence	5,017	111	3,069	101	17	127
**Musculoskeletal diseases**	**2,427,408**				**9,083**	
Other back problems	1,294,680	1	247,383	2	3,880	18
All other musculoskeletal diseases	630,910	7	196,122	5	4,175	17
Cervicalgia	252,100	18	61,970	25	89	97
Pain in foot	168,750	24	71,302	21	15	129
Osteoarthritis	45,411	44	20,771	52	479	58
Other knee disorders	16,432	72	6,521	79	356	67
Other shoulder disorders	15,165	77	6,584	78	68	101
Rheumatoid arthritis	3,960	116	1,399	114	21	125
**Signs, symptoms, ill-defined conditions**	**1,234,823**				**12,423**	
All other signs and symptoms	758,270	6	329,863	1	10,574	8
Respiratory, chest signs and symptoms	273,022	14	163,807	8	756	47
Abdomen, pelvis signs and symptoms	203,531	21	124,481	13	1,093	38
**Neurological conditions**	**1,007,643**				**6,251**	
Organic sleep disorder	845,886	4	172,142	6	525	56
Chronic pain	92,779	29	27,871	39	298	73
All other neurological conditions	47,406	41	18,228	56	4,581	16
Other mononeuritis, upper / lower limbs	13,046	84	5,971	80	29	119
Epilepsy	5,827	106	1,881	109	709	50
Multiple sclerosis	2,476	124	504	129	101	94
Parkinson's disease	223	148	54	147	8	138
**Sensory organ diseases**	**545,237**				**828**	
Refraction, accommodation	252,513	17	206,459	4	2	147
All other sensory organ diseases	193,346	22	119,617	14	800	44
Hearing disorder	82,075	32	48,695	27	6	141
Glaucoma	15,510	76	10,321	70	5	144
Cataracts	1,793	128	963	119	15	129
**Skin diseases**	**395,364**				**3,119**	
All other skin diseases	272,738	15	152,833	10	3,056	22
Sebaceous gland disease	73,933	34	41,377	29	27	121
Contact dermatitis	48,693	40	35,973	32	36	113
**Infectious and parasitic diseases**	**219,126**				**6,212**	
All other infectious and parasitic diseases	92,425	30	62,288	24	4,996	15
Unspecified viral infection	31,754	52	29,171	38	45	109
Tinea skin infection	26,613	56	20,937	51	0	150
COVID-19	26,418	58	23,173	47	258	76
Diarrheal disease	18,140	69	15,739	61	651	53
Sexually transmitted disease (STD)	15,895	74	11,807	67	116	91
Chlamydia	5,656	108	5,034	89	10	134
Hepatitis B, C	1,261	131	539	127	2	147
Tuberculosis	340	143	169	141	22	124
Intestinal nematode infection	330	144	282	136	0	150
Malaria	158	151	57	146	70	100
Tropical cluster	73	155	41	151	2	147
Bacterial meningitis	63	156	20	156	40	111
**Respiratory infections**	**390,987**				**2,682**	
Upper respiratory infection	311,946	13	229,755	3	449	62
Lower respiratory infection	49,347	39	31,959	33	2,180	28
Otitis media	29,694	53	23,243	46	53	105
**Respiratory diseases**	**255,014**				**3,462**	
Allergic rhinitis	113,649	27	38,889	31	11	133
All other respiratory diseases	46,858	42	27,340	41	2890	23
Asthma	33,632	51	15,154	62	274	75
Chronic sinusitis	24,122	61	16,678	60	36	113
Deviated nasal septum	16,149	73	8,806	72	213	80
Chronic rhinitis	11,908	86	8,751	73	0	150
Chronic obstructive pulmonary disease	8,696	95	7,428	74	38	112
**Genitourinary diseases**	**383,433**				**4,674**	
All other genitourinary diseases	186,694	23	87,963	19	1,730	30
Female genital pain	59,224	35	24,829	44	48	108
Menstrual disorder	36,248	47	22,053	48	336	69
UTI, cystitis	25,557	59	19,058	54	211	81
Other breast disorders	24,340	60	12,591	66	304	72
Vaginitis, vulvitis	18,136	70	13,515	65	0	150
Kidney stones	15,632	75	6,634	77	454	61
Nephritis, nephrosis	14,269	79	5,154	88	1,557	31
Benign prostatic hypertrophy	3,333	118	2,068	108	34	118
**Digestive diseases**	**271,707**				**15,407**	
All other digestive diseases	129,648	25	67,054	22	8,814	9
Esophagus disease	54,746	37	31,932	34	690	51
Other gastroenteritis and colitis	46,466	43	30,455	36	1,511	32
Constipation	21,229	66	14,296	64	86	98
Inguinal hernia	10,092	90	3,929	96	168	84
Appendicitis	7,321	100	2,829	104	3,463	21
Peptic ulcer disease	1,352	130	807	121	321	71
Cirrhosis of liver	853	135	209	139	354	68
**Maternal conditions**	**203,467**				**54,348**	
Pregnancy complications, delivery	121,660	26	24,673	45	33,961	3
All other maternal disorders	44,321	45	11,736	68	5,978	12
Delivery	21,631	65	9,033	71	12,626	6
Ectopic pregnancy, miscarriage, abortion	10,173	89	4,420	93	456	60
Puerperium complications	5,682	107	3,014	102	1,327	35
**Headache**	**208,826**				**988**	
Headache	208,826	20	100,169	17	988	40
**Cardiovascular diseases**	**185,229**				**7,721**	
All other cardiovascular diseases	88,514	31	42,079	28	3,488	20
Essential hypertension	75,545	33	39,104	30	432	64
Cerebrovascular disease	9,687	93	2,221	107	2,622	25
Ischemic heart disease	7,647	98	2,980	103	782	46
Inflammatory	3,133	120	1,520	113	391	66
Rheumatic heart disease	703	137	629	125	6	141
**Other neoplasms**	**89,143**				**1,763**	
All other neoplasms	35,225	49	21,803	49	1,135	37
Benign skin neoplasm	23,806	63	18,841	55	10	134
Neoplasm of uncertain behavior of skin	13,454	82	11,381	69	0	150
Lipoma	9,899	92	5,805	83	55	104
Uterine leiomyoma	6,759	103	3,117	100	563	55
**Endocrine disorders**	**65,946**				**461**	
Hypothyroidism	14,663	78	7,194	75	27	121
Other thyroid disorders	14,193	80	5,553	85	196	82
Testicular hypofunction	14,056	81	5,692	84	6	141
All other endocrine disorders	9,491	94	4,822	92	218	79
Polycystic ovarian syndrome	7,082	101	3,952	95	9	136
Unspecified disorder of pituitary gland	6,461	104	3,565	99	5	144
**Malignant neoplasms**	**50,547**				**6,365**	
All other malignant neoplasms	7,957	96	1,209	116	1,388	34
Lymphoma and multiple myeloma	7,583	99	649	124	841	43
Breast cancer	5,610	109	511	128	434	63
Melanoma and other skin cancers	5,151	110	2,226	106	75	99
Leukemia	4,652	112	287	135	1,486	33
Testicular cancer	4,497	113	653	123	226	77
Colon, rectum cancers	4,412	115	324	134	656	52
Brain cancer	3,017	121	224	137	477	59
Thyroid cancer	2,006	125	452	131	107	92
Mouth, oropharynx cancers	1,164	132	138	142	94	96
Prostate cancer	1,125	133	220	138	52	106
Cervix uteri cancer	974	134	498	130	15	129
Trachea, bronchus, lung cancers	612	140	83	143	133	87
Stomach cancer	549	141	34	153	98	95
Pancreatic cancer	299	145	47	150	127	88
Ovarian cancer	268	146	52	148	21	125
Liver cancer	237	147	38	152	23	123
Corpus uteri cancer	167	150	31	154	14	132
Bladder cancer	156	152	48	149	35	116
Esophagus cancer	111	154	9	157	63	102
**Metabolic and immune disorders**	**48,247**				**494**	
Lipoid metabolism disorder	26,861	55	19,750	53	51	107
Other metabolic disorders	10,641	88	5,840	82	333	70
Gout	7,871	97	3,991	94	8	138
Immune disorder	2,874	123	952	120	102	93
**Nutritional disorders**	**80,982**				**107**	
Overweight, obesity	52,329	38	27,539	40	42	110
All other nutritional disorders	28,454	54	21,705	50	36	113
Protein-energy malnutrition	199	149	79	144	29	119
**Blood disorders**	**43,712**				**871**	
All other blood disorders	13,244	83	5,908	81	486	57
Other non-deficiency anemias	12,193	85	6,910	76	220	78
Iron deficiency anemia	10,873	87	4,949	90	122	89
Hereditary anemia	6,767	102	5,247	86	35	116
Other deficiency anemias	635	138	398	133	8	138
**Oral conditions**	**38,650**				**1,072**	
All other oral conditions	35,948	48	25,521	42	1,063	39
Dental caries	1,877	127	1,790	110	0	150
Periodontal disease	825	136	779	122	9	136
**Congenital anomalies**	**28,502**				**862**	
All other congenital anomalies	23,929	62	14,493	63	585	54
Congenital heart disease	2,898	122	1,307	115	122	89
Other circulatory anomalies	1,675	129	623	126	155	86
**Diabetes mellitus**	**21,099**				**733**	
Diabetes mellitus	21,099	67	5,191	87	733	49
** Conditions arising during perinatal period ^e^**	**1,201**				**79**	
All other perinatal anomalies	627	139	401	132	62	103
Low birth weight	447	142	186	140	0	150
Birth asphyxia and birth trauma	127	153	69	145	17	127

Abbreviations: No., number; UTI, urinary tract infection; STD, sexually transmitted disease.

a Burden of disease major categories and burden of disease-related conditions modified from those defined in Global Burden of Disease Study. ^3 , 4^

b Medical encounters include total hospitalizations and ambulatory visits for the condition (with no more than 1 encounter per individual per day per condition).

c Individuals with at least 1 hospitalization or ambulatory visit for the condition.

d Rank based on the number of encounters, individuals affected, or hospital bed days in the respective columns within the listing of 157 burden-related disease conditions. For hospital bed days, there were 8 conditions with the rank of 150 (0); 14 other conditions had tied rankings.

e Conditions affecting newborns erroneously coded on service member medical records.

### Individuals affected, by category

In 2024, the 10 categories of conditions that affected the most service members were signs, symptoms, and other ill-defined conditions (all other signs and symptoms), musculoskeletal diseases (other back problems, all other musculoskeletal diseases), respiratory infections (upper respiratory infections) sensory organ diseases (refraction / accommodation), neurological conditions (organic sleep disorders), injuries (knee, arm / shoulder), respiratory diseases, and skin diseases (all other skin diseases). COVID-19 affected 23,173 ACSMs and ranked forty-seventh for members affected, a considerable decrease in rank from thirty-fifth in 2023.

### Hospital bed days, by condition

**FIGURE 3.**
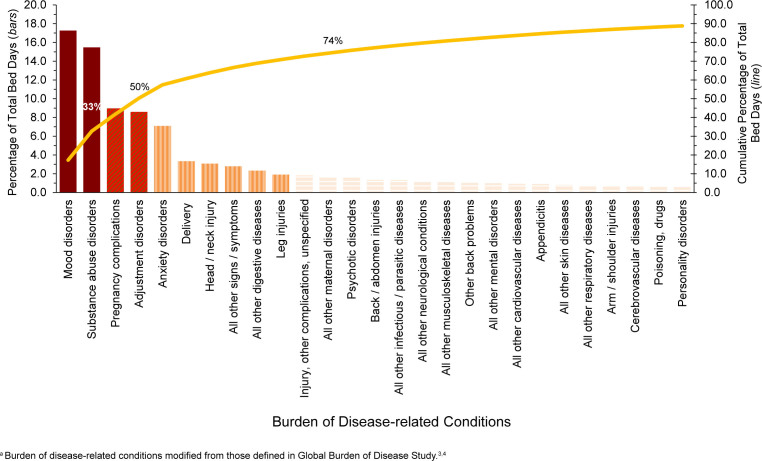
Percentage and Cumulative Percentage Distribution, Burden of Disease-related Conditions ^a^ that Accounted for the Most Hospital Bed Days, Active Component, U.S. Armed Forces, 2024

Mood and substance abuse disorders accounted for nearly one-third (32.7%) of all hospital bed days in 2024 **(Figure 3)** . Four mental health disorders (mood, substance abuse, adjustment, anxiety) and 2 maternal conditions (pregnancy complications, delivery) together accounted for almost two-thirds (60.7%) of all hospital bed days **(Table** and **Figure 3)** . About 11.3% of all hospital bed days were attributable to injury. COVID-19 accounted for 0.1% of total hospital bed days among ACSMs **(Table)** .

### Relationships between health care burden indicators

There was a strong positive correlation between numbers of medical encounters attributable to various medical conditions with numbers of individuals affected by those conditions (r=0.85) (data not shown). The 3 leading causes of medical encounters were among the 5 medical conditions that most affected individuals **(Table)** , while weak-to-moderate positive relationships were detected between numbers of hospital bed days attributable to conditions with numbers of individuals affected by those conditions (r=0.20), or numbers of medical encounters related to a medical condition (r=0.40). For example, substance abuse disorders and labor and delivery ranked high in terms of total bed days, these conditions affected relatively few ACSMs in 2024.

## Discussion

This *MSMR* report provides the most recent data available for major disease classification and analysis comparable to previous reports. The total number of conditions reported in 2024 increased by 0.8% compared to 2023, and medical encounters increased by 1.3%. The numbers of affected individuals and hospital bed days decreased, however, by 4.4% and 2.9%, respectively. While numbers of individuals affected and hospital bed days decreased in 2024, the major diseases and conditions observed in this analysis are consistent with previous *MSMR* reports on the morbidity and health care burdens of the U.S. military.

Compared to 2023, both numbers of medical encounters and hospital bed days decreased for 5 major categories—mental health disorders, musculoskeletal diseases, respiratory diseases, maternal conditions, and blood disorders—while in the remaining categories, changes in numbers of medical encounters and hospital bed days were inconsistent. Injuries, mental health disorders, and musculoskeletal disorders were the categories in 2024 associated with the most medical encounters, highest numbers of affected service members, and greatest numbers of hospital bed days.

Only 9 of the 157 medical conditions that comprise this report, or just 5.7% of the listed conditions, accounted for slightly more than half (51.6%) of all illness- and injury-related medical encounters: 2 anatomical, site-defined injuries (knee, arm / shoulder), 3 mental health disorders (anxiety, adjustment, mood disorders), 2 musculoskeletal conditions (other back problems, all other musculoskeletal diseases), 1 sign, symptom or ill-defined condition (all other signs and symptoms), and 1 neurological condition (organic sleep disorders).

The pattern of illness and injury among U.S. ACSMs is distinct from other population groups, with different demographic distributions and occupational hazards. Injuries, mental disorders, and musculoskeletal diseases are identified in the literature as among the leading causes of morbidity and disability among service members throughout military history, affecting readiness and health care provision. ^8 - 10^ A previous study reported that injuries were the single leading cause of death, disability, hospitalization, outpatient visits, and manpower loss among U.S. military service members. ^8^ Exposure to intense physical demands during training and in operational environments increases risk of musculoskeletal injury, which contributes to significant morbidity among military personnel. ^11^ Due to lifestyles that can be influenced by operational conditions, multiple combat missions, and familial separations, among other factors, a number of mental disorders including occupational stress, depression, and suicide are common among military personnel. ^9^ Some studies have reported significant associations between major depressive disorder and deployment. ^10^

Reporting on the burden of disease and injury includes reliable quantification of their physical and psychosocial health impacts, as well as risk factors, that can provide valuable information about a population's health status, for optimal resource allocation for prevention and treatment. Accurate estimates can be used to predict expected health care use and costs, prioritize effective interventions, and evaluate their impacts and cost effectiveness. ^6^ Current, accurate information on the scale of health disorders among service members, groups at significant risk, and trends in their health statuses over time are critical for policy-makers and commanders.

Preventing injuries and illnesses in service members requires not only routine injury and disease monitoring, but informed, pervasive understanding of the link between health-related factors and disease occurrence, a comprehensive medical surveillance system for successful prevention programs, and data-driven research prioritization. These surveillance, analysis, and reporting efforts can culminate in effective partnerships between commanders, policy-makers, and service members for direct actions to prevent disease and injury. ^8 , 11^

With psychosocial factors shown to be implicated in increased risk of back pain, for example, addressing related health care issues holistically, rather than divided among discrete categories, would be beneficial. ^12 , 13^ Integrated approaches to care not only address identified burdens of medical conditions but their associated risk factors. The unique health challenges of the military population share risk factors and medical conditions with the civilian population, with the added complexities of service experience and the nature of combat. ^15^

## References

[1] Hernandez JBR , Kim PY . Epidemiology morbidity and mortality . In: StatPearls [internet] . Updated Oct. 3, 2022 . StatPearls Publishing ; 2024 . https://www.ncbi.nlm.nih.gov/books/nbk547668 31613448

[2] Roser M , Ritchie H , Spooner F . Burden of Disease . 2024 . Accessed Aug. 14, 2025 . https://our-worldindata.org/burden-of-disease

[3] World Health Organization . The Global Burden of Disease: 2004 Update . World Health Organization ; 2008 . Accessed Aug. 14, 2025 . https://www.who.int/publications/i/item/9789241563710

[4] Murray CJL , Lopez AD , eds. The Global Burden of Disease: A Comprehensive Assessment of Mortality and Disability from Diseases, Injuries, and Risk Factors in 1990 and Projected to 2020 . Harvard University Press ; 1996 : 120 - 122 .

[5] Murray CJL . The Global Burden of Disease Study at 30 years . Nat Med . 2022 ; 28 ( 10 ): 2019 - 2026 . doi: 10.1038/s41591-022-01990-1 36216939

[6] Devleesschauwer B , Maertens de Noordhout C , Smit GS , et al . Quantifying burden of disease to support public health policy in Belgium: opportunities and constraints . BMC Public Health . 2014 ; 14 : 1196 . doi: 10.1186/1471-2458-14-11967 25416547 PMC4246467

[7] World Health Organization . WHO Methods and Data Sources for Global Burden of Disease Estimates 2000-2019 . World Health Organization ; 2020 . Accessed Jun. 4, 2024 . https://www.who.int/docs/default-source/gho-documents/global-health-estimates/ghe2019_daly-methods.pdf

[8] Jones BH , Perrotta DM , Canham-Chervak ML , Nee MA , Brundage JF . Injuries in the military: a review and commentary focused on prevention . Am J Prev Med . 2000 ; 18 ( 3 suppl 1 ): 71 - 84 . doi: 10.1016/s0749-379(99)00169-5 10736543

[9] Moradi Y , Dowran B , Sepandi M . The global prevalence of depression, suicide ideation, and attempts in the military forces: a systematic review and meta-analysis of cross sectional studies . BMC Psychiatry . 2021 ; 21 ( 1 ): 510 . doi: 10.1186/s12888-021-03526-2 34654386 PMC8520236

[10] Packnett ER , Elmasry H , Toolin CF , Cowan DN , Boivin MR . Epidemiology of major depressive disorder disability in the US military: FY 2007-2012 . J Nerv Ment Dis . 2017 ; 205 ( 9 ): 672 - 678 . doi: 10.1097/nmd.0000000000000692 28640037

[11] Lovalekar M , Hauret K , Roy T , et al . Musculoskeletal injuries in military personnel: descriptive epidemiology, risk factor identification, and prevention . J Sci Med Sport . 2021 ; 24 ( 10 ): 963 - 969 . doi: 10.1016/j.jsams.2021.03.016 33824080

[12] To D , Rezai M , Murnaghan K , Cancelliere C . Risk factors for low back pain in active military personnel: a systematic review . Chiropr Man Therap . 2021 ; 29 ( 1 ): 52 . doi: 10.1186/s12998-021-00409-x PMC8719410 34969400

[13] Meints SM , Edwards RR . Evaluating psychosocial contributions to chronic pain outcomes . Prog Neuropsychopharmacol Biol Psychiatry . 2018 ; 87 ( pt b ): 168 - 182 . doi: 10.1016/j.pnpbp.2018.01.017 29408484 PMC6067990

[14] Alruwaili A , Khorram-Manesh A , Ratnayake A , Robinson Y , Goniewicz K . Supporting the front-lines: a scoping review addressing the health challenges of military personnel and veterans . Healthcare (Basel) . 2023 ; 11 ( 21 ): 2870 . doi: 10.3390/healthcare11212870 37958012 PMC10648823

